# Correlation between visual field index and quality of life in glaucoma patients: a new tool to screen quality of life perception?

**DOI:** 10.3389/fmed.2023.1214007

**Published:** 2023-09-04

**Authors:** Gemma Caterina Maria Rossi, Giovanni Milano, Annalisa De Silvestri, Lorenzo Savini, Chiara Bosi, Giulia Gambini, Paolo Rama

**Affiliations:** ^1^La Struttura Complessa Oculistica, Fondazione IRCCS Policlinico San Matteo, Pavia, Italy; ^2^Ambulatorio Glaucoma, ASST Bergamo Est, Ospedale Locatelli, Piario, Bergamo, Italy; ^3^Dipartimento Scienze Clinico-Chirurgiche, Diagnostiche e Pediatriche, Università degli Studi di Pavia, Pavia, Italy; ^4^SSD Biostatistica e Clinical Trial Center, Direzione Scientifica, Fondazione IRCCS Policlinico San Matteo, Pavia, Italy; ^5^Facoltà di Medicina e Chirurgia, Università degli Studi di Pavia, Pavia, Italy

**Keywords:** visual field index, quality of life, glaucoma, screening, gender, NEI-VFQ 25, GSS, questionnaire

## Abstract

**Purpose:**

To evaluate the correlation between the visual field index (VFI) and vision-related quality of life (QoL) considering several confounding variables that may have a positive or negative effect.

**Methods:**

We conducted a cross-sectional, mono-centric study on glaucoma patients. Quality of life was examined with the NEI-VFQ 25 and the Glaucoma Symptom Scale (GSS). The visual field was examined with the Humphrey Field Analyzer. The variables considered were age, gender, comorbidities, years (at diagnosis and duration of the illness), treatment and related active principles, intraocular pressure, and visual acuity. The analysis was performed on both the better and the worse eye. The linear regression univariate analysis and the multivariate analyses were performed.

**Results:**

In total, 193 patients enrolled in the study. The mean age was 70.8 ± 10.4 years. The mean follow-up period since diagnosis 11.4 ± 9.2 years. Approximately 50% of the patients suffered from primary open angle glaucoma (POAG) and 45% were on monotherapy. The mean VFI was 81.3 ± 26. Regarding QoL, the NEI-VFQ total mean was 80.4 ± 17.8 and the GSS total score was 77.2 ± 21. Regarding NEI-VFQ 25, the single linear regression analysis found the following relations: age at time of visit (*r* = −0.30, *p* = 0.016), years of illness (*r* = −0.32, *p* = 0.020), the minimum and maximal visual acuity (*r* = 2.04 and *r* = 3.96, *p* < 0.001), the IOP min (*r* = 1.13, *p* = 0.002) and max (*r* = −0.52, *p* = 0.017), and the number of previous surgeries (*r* = −3.94, *p* < 0.001). The multivariate analysis found the following relations: gender (*r* = 5.13, *p* = 0.019), visual acuity max (*r* = 3.16, *p* < 0.001), and previous surgeries (*r* = −1.80, *p* = 0.032). Regarding GSS, the single linear regression analysis found relations with visual acuity (*r* = 2.37, *p* < 0.001), VFI (*r* = 0.41, *p* < 0.001), previous surgeries in the eye considered (*r* = −7.27, *p* < 0.001), and number of instillations (*r* = −3.67, *p* = 0.031). Data confirmed that a higher VFI has a positive impact on the score of both the NEI-VFQ 25 (*r* = 0.22, *p* = < 0.001) and the GSS questionnaire (*r* = 0.36, *p* < 0.001).

**Conclusions:**

The study demonstrated a correlation between the VFI and QoL of patients and their visual and non-visual ocular symptoms and function both in the worst and in the better eye, even when accounting for several clinical and demographic confounding variables. Our data support that the visual field index is an important metric instrument in the follow up of patients with glaucoma.

## Introduction

Glaucoma is a chronic disease that affects visual function and negatively impacts on vision-related quality of life (VRQoL). In recent years, the role of VRQoL in the treatment of glaucoma has become increasingly important, to the point that the European Glaucoma Society (EGS), in their latest guidelines, defines that the purpose of glaucoma care is the promotion of wellbeing and quality of life with the aim to maintain visual function and quality of life (QoL) within a sustainable health care system (1). The main aim of glaucoma specialists is to detect the disease at an early stage and prevent any further progression to preserve patients' independence.

QoL and wellbeing are subjective and abstract parameters: they are difficult to evaluate and compare because they depend on and are influenced by individual characteristics. The residual visual function, the psychological impact of having a chronic progressive sight-threatening disease, and the side effects of treatment impact and influence patient QoL in different ways.

Wellbeing is the experience of health, happiness, and prosperity. It includes having good mental health, good physical health and function, high life satisfaction, a sense of meaning or purpose, positive relationships, affects, community, social support, and financial stability. More generally, wellbeing is just feeling well, but it is not so easy to evaluate, measure, maintain, or improve.

QoL and wellbeing inevitably overlap, and the terms are often used interchangeably since they are both subjective assessments and refer to psychological states, but they are different and should be treated as separate concepts (2).

In short, QoL refers to the cognitive appraisal which a patient makes about the impact their health has on their daily life, whilst wellbeing concerns a patient's emotional response to their illness, its treatment, and their future (2).

In 2012, an international consensus (3) discussed how to measure wellbeing and have provided an excellent starting point for taking this vital work forward, but to date there remains much to be done in relation to defining dimensions and developing assessment tools.

On the contrary, QoL measurement has been considered one of the most difficult and challenging areas faced by health care professionals. Despite this, there are still some tools for patient self-report assessment. In-practice quantitative fixed-response measures such as questionnaires are normally used in a clinical setting over the course of several years. The questionnaires measure QoL with two possible approaches: generic or condition-specific assessment.

The vision-related QoL in glaucoma is measured with both generic and specific validated questionnaires that can measure changes over time; one of the most frequently used questionnaires is the 25-item National Eye Institute Visual Function Questionnaire (NEI-VFQ 25) (4). The NEI-VFQ 25 is a generic vision-related QoL questionnaire which has demonstrated its usefulness, sensitivity, and specificity in the investigation of patient's quality of life and in relation to visual field loss and is now available in several languages. Another questionnaire, specifically designed to quantify the impact of symptoms related to glaucoma treatment (glaucoma-related QoL), is the Glaucoma Symptoms Scale (GSS) (5).

The visual field examination is the test to diagnose, stage, and follow glaucoma.

For Humphrey perimeters, there are three indexes used to evaluate the visual field loss and its progression over time: the mean deviation (MD), the pattern standard deviation (PSD), and the visual field index (VFI). MD represents the degree of deviation of the field from average value, in the age-matched normal population; PSD represents irregularities in the field, such as localized field defects; and VFI is a staging index that corresponds to ganglion cell loss (6).

In literature, the relationship between quality of life and visual field index has not often been addressed. There are only two studies reporting some sort of correlation (7, 8). Sawada et al. (7) examined the significance of the relationship between QoL and visual field, and compared the strength of correlations between MD and VFI: they found that the correlation coefficients of the VFI were slightly higher than those of MD overall. Lee examined the relationship between QoL and VFI and found a poorer QoL (Glaucoma Quality of Life−15 questionnaire) score and a lower visual field index (8).

Several publications have examined the association between quality of life and visual field, mainly considering MD, but it is well known that this parameter is not specific for the glaucoma defect since it may also be affected by global defects like cataract (6).

For this reason, we choose to evaluate VFI as percentage summarizing the overall visual field status compared to age-adjusted visual fields, since this parameter emphasizes the importance of the central field and is less affected by media opacities (6).

The purpose of the study is to evaluate the correlation between the visual function, as determined by the VFI, and both the general and glaucoma-specific QoL in glaucoma patients, because there are few studies that have considered this parameter to determine the quality of life even though this parameter is more useful than MD in the evaluation of glaucomatous pathology.

The study also considers some confounding variables that may have positive or negative effects on quality of life other than VFI and its correlation to it.

## Methods

This was a single center, observational study carried out at the University Eye Clinic of Pavia in accordance with the Declaration of Helsinki after approval by the Local Ethics Committee of the Fondazione IRCCS Policlinico San Matteo of Pavia (prot. 2014000576, proc, P-20140031162, study protocol OCU-GLC-2014). The study has been conducted according to the recommendations of the Helsinki declaration (revision 2000, Edimbourg) and to the Italian Good Clinical Practice legislation (DM 15 Luglio 1997 and modifications).

Signed informed consent was obtained from all individual participants included in the study.

Inclusion criteria were having a diagnosis of either primary open angle glaucoma (POAG), normal tension glaucoma (NTG), glaucoma suspect (GS), angle closure glaucoma (ACG), pseudoexfoliative glaucoma (PEX), pigmentary glaucoma (PG), or uveitic glaucoma (UG). Exclusion criteria of the study was the inability, either because of physical or mental disabilities, of the patient to properly complete the questionnaire, either independently or with the help of someone reading questions for them.

The inclusion/exclusion criteria were verified after careful examination of patients' medical charts. If eligible, at his/her first clinical visit after study start, the patient was informed of the protocol and his/her consent required in written form.

The diagnoses of the various conditions were based on the EGS guidelines (1) and are as follows: POAG: the concomitant presence of characteristic acquired glaucomatous damage and/or retinal nerve fiber layer changes, glaucomatous visual field defect, and elevated intraocular pressure (IOP) without treatment; NTG: optic nerve damage and visual field defects typical of glaucoma and normal IOP without treatment; GS: visual field and /or optic disc and /or nerve fiber layer normal or suspicious with at least one being suspicious, and IOP normal or increased; ACG: elevated IOP, peripheral anterior synechiae, and glaucomatous visual field and optic nerve; PEX: elevated IOP, glaucomatous visual field and optic nerve, and dandruff-like exfoliation material on the pupil border and on the surface of the anterior lens capsule; PG: dispersion of iris pigment and glaucomatous visual field and optic nerve; and UG: acute IOP elevation due to uveitic inflammation and glaucomatous visual field and optic nerve.

For each patient, medical and clinical history were collected. Information gathered included age, gender, diagnosis, the date of diagnosis, reported family history of glaucoma, concomitant systemic diseases (diabetes, hypercholesterolemia, hypertension, arthrosis or arthritis, or cardiopathy), systemic treatments, topical glaucoma therapy (when eye drops were first started, when the ongoing treatment started, the type of treatment, active principles contained, and daily number of instillations and bottle used), and previous eye surgery or lasers and their dates.

The clinical examination recorded data about the vision and eye health. Visual acuity was measured with a Snellen chart, gaining information regarding the presence of refractive errors and their values. The anterior segment of the eye was examined and the lens evaluated (transparent, sclerosis, cataract, or pseudophachy). The fundus examination was performed with particular attention to the abnormalities or asymmetries in the morphology of the head of the optic nerve (normal, excavated, or tilted) and the cup disc ratio. The IOP was measured with the Goldmann tonometer. If not previously present, ultrasound pachymetry was measured.

Patients before the visit were asked to perform the visual field test with the Humphrey Field Analyzer (SITA fast 24–2 program). All the information regarding the visual field test of each eye were collected: GHT (normal, borderline or pathological), VFI (from 100% VFI = normal fields to 0% VFI = blind fields), MD, and PSD.

Patients completed two vision-related QoL questionnaires: the NEI -VFQ 25 (4) and the GSS (5). Both questionnaires are validated for self-administration; the ophthalmologist explained how to answer the questionnaires, but each patient filled them out independently.

The NEI-VFQ 25 (4) is a questionnaire that consists of 25 questions related to visual function in everyday life. It is divided into three major categories: 1, General health (1 item); 2, Quality of vision (9 items); and 3, Vision-related quality of life (15 items) (4).

The questionnaire is composed of multiple-choice questions, where the answers ranged from the best health, vision, and function (with a score of 100) to the poorest ones (with a score of 0). The scores of each subscale and the total mean score were calculated using the decoding system validated for this questionnaire (4).

The Glaucoma Symptoms Scale questionnaire is composed of 10 ocular disorders and investigating their complaints from the previous 4 weeks (5). Each item was to be answered for both eyes, with 100 points showing the absence of symptoms, while 0, 25, 50 or 75 relating how bothersome this side effect is for the patient. The 10 ocular complaints are divided into six items related to non-visual ocular symptoms, and four items of visual ocular complaints. The symptoms considered are burning, lacrimation, dryness, itching, ocular pain, blurred vision, foreign body sensation in the eye, difficulties seeing in daylight, difficulties seeing in dark places, and perception of halos around lights. The scores for each subscale were calculated using the decoding system validated for this questionnaire (5).

### Statistical analysis

The primary endpoint was to evaluate the association between QoL and VFI.

Both questionnaires were decoded following the instructions of the respective validation studies (4, 5).

The secondary endpoint was to consider other confounding variables that may have positive or negative effects on the quality of life other than VFI and their association with it. These variables are age, gender, comorbidities, years at the time of the visit and at the time of diagnosis, duration of the illness, treatment and related active principles, number of bottles prescribed and number of instillations per day, previous surgery, IOP min and max, and visual acuity min and max. The analysis was performed on both the better and the worse eye, to see the extent of association to the quality of life in each case.

#### Power consideration

According to the rule of thumb, of 10/20 cases for each variable included in the models, 193 patient models with up to 10 covariates can be fitted.

The primary endpoint was analyzed, with fitting linear regression models having NEI-VFQ 25 mean score or GSS as dependent variables and mean, minimum, or maximum VFI as independent variables.

To analyze secondary endpoints, multivariable models were fitted, including the abovementioned clinic demographic covariates that are not collinear and that have a *p* < 0.20 in univariate analyses. This is done to determine, since this is an observational study, if the association between the VFI max and NEI-VFQ 25 score could be biased by other confounding variables or if the association is still significant.

Regarding the GSS symptoms and function scales, the population studied is different since there are two different scores, one for each eye, so the VFI is not considered as mean, minimum, and maximum value, but separately. Therefore, in this statistical model, the subjects of the analysis are the eyes and not the patients: in this case, the analyses are clustered per patients.

#### Sensitivity analyses

Quality of life scores were also calculated for a sensitivity analysis with the item response theory graded response model (IRT-GRM) that avoids the Lickert scale bias. In the GRM, item responses are categorical and ordered, allowing the ordered categories to vary between items. In the GRM, each item is modeled with its own discrimination parameter and cut points that identify boundaries between the ordered outcomes. Test characteristic curve (TCC; plot of the expected score against the latent trait) was used to graphically compare NEI-VFQ 25 and GSS scores decoded following the instructions of the respective validation studies (4, 5) vs. predicted score from the IRT-GRM. Furthermore, as sensitivity analyses, the latent traits of both questionnaires predicted using an empirical Bayes estimator were entered as dependent variables in the multivariate models developed for QoL scores decoded following the instructions of the respective validation studies (4, 5).

## Results

One hundred ninety-three patients (103 females, 53.1%), followed at the Glaucoma center of the University Eye Clinic of the Fondazione I.R.C.S.S. Policlinico San Matteo of Pavia, were enrolled.

The mean age at the time of the visit was 71 years old, with a standard deviation of 10.4 years.

The average age at which patients were diagnosed with glaucoma was 59 ± 11.9 years. The mean number of years spent with glaucoma was 11.4 ± 9.2 years (Table 1).

**Table 1:** Demographic and clinical data (N, number; SD, standard deviation; IQR, interquartile range; confirmed glaucoma: primary open angle glaucoma + normal tension glaucoma + angle closure glaucoma + pseudoexfoliative glaucoma + pigmentary glaucoma + uveitic glaucoma).

**Variable**	**Overall group**		**Confirmed glaucoma**	**Suspected glaucoma**	***p*-value**
	**Mean** ±**SD**	**Min-Max**	**Mean** ±**SD**	**Mean** ±**SD**	
Age at time of visit (years)	70.8 ± 10.4	19.2–89.5	72 ± 9.5	66 ± 12	< 0.01
Age at time of diagnosis (years)	59.5 ± 11.9	17–82	60 ± 11.4	56 ± 13	< 0.02
Length of illness (years)	11.4 ± 9.2	1–40	12 ± 9.2	11 ± 9.3	0.646
**Gender**	***N*** **(%)**		***N*** **(%)**		
Female	103 (53.1)		73 (49)	26 (62)	0.150
**Diagnosis**					
Primary open angle glaucoma, POAG	221 (58.2)				
Glaucoma suspect, GS	84 (22.1)				
Angle closure glaucoma, ACG	25 (6.6)				
Pseudoexfoliative glaucoma, PEX	28 (7.4)				
Normal tension glaucoma, NTG	12 (3.2)				
Pigmentary glaucoma, PG	2 (0.5)				
Uveitic glaucoma, UG	8 (2.1)				
Familiarity for glaucoma	14 (8.9)		4 (3.3)	10 (28.5)	< 0.01
**Systemic co-morbidities**					
Diabetes	33 (20.1)		27 (21.4)	6 (17)	0.578
Hypertension	109 (61.6)		85 (62)	21 (58)	0.684
Hypercholesterolemia	34 (20.4)		28 (22)	5 (13.5)	0.246
Arthritis/Arthrosis	7 (4.3)		4 (3)	3 (8.5)	0.173
Myocardial ischemic cardiopathy	19 (11.6)		15 (12)	4 (11)	0.884
	**Mean** ±**SD**	**Median[IQR]**	**Mean** ±**SD**	**Mean** ±**SD**	
Number of concomitant systemic pathologies	1.7 ± 1.3	2 [1–2]	1.7 ± 1.3	1.58 (1.2)	0.375
**Visual field (VF)**	**Mean** ±**SD**	**Min–Max**	**Mean** ±**SD**	**Mean** ±**SD**	
Visual field index (VFI) (%)	81.3 ± 26	0–100	77 ± 27.6	96.5 (6.4)	< 0.01
Mean deviation (MD) (dB)	6.7 ± 8.1	−32.2 ± 4.9	−8± 8.5	−1.5 (2.8)	< 0.01
Pattern standard deviation (PSD) (dB)	4.7 ± 3.7	1.1–14.8	5 ± 3.7	2 (2)	< 0.01
**Visual acuity (decimals)**	0.7 ± 0.3	0–1.0	7.3 ± 3.4	8.7 ± 2	< 0.01
**Intraocular pressure (IOP) (mmHg)**	15.2 ± 5.1	6–50	15± 5.4	16.5 ± 3.5	< 0.01

Table 1 shows the distribution of the type of glaucoma in the studied group: primary open angle glaucoma was present in 58% of the patients, while 22% were glaucoma suspects.

Some differences have been observed between suspected and confirmed glaucoma patients.

Confirmed glaucoma patients were older and the time of diagnosis was later (Table 1). Of the glaucoma suspects, 28% had a family history of glaucoma.

Most patients suffered from hypertension (109, 62%), followed by hypercholesterolemia (34, 20%) and diabetes (33, 20%). The median number of concomitant comorbidities in the examined population was two, without any difference between groups (Table 1).

Regarding actual topical therapy, most patients were in therapy with prostaglandin analogs and beta blockers, alone or in association (Table 2). Most patients (45.4%) were on monotherapy as single drug or fixed combination; only 3% used three bottles a day. Thirty-nine percent of the patients used the drops three times a day, 37% once a day, and 17% twice (Table 2).

**Table 2:** Topical therapies and previous surgeries (N, number; SD, standard deviation; IQR, interquartile range).

**Topical therapy**	**Overall group**		**Confirmed glaucoma**	**Suspected glaucoma**	***p*-value**
	***N*** **(%)**		***N*** **(%)**	***N*** **(%)**	
Beta blocker, BB	211 (74)		174 (76)	33 (30)	0.246
Prostaglandin analogs, PG	226 (78.2)		191 (82)	33 (37)	0.201
Topical carbonic anhidrase inibitors, CAI	134 (49.5)		115 (52)	17 (15)	0.157
α2- agonists	22 (8.7)		18 (82)	4 (18)	0.938
	**Mean** ±**SD**	**Median [IQR]**	**Mean** ±**SD**	**Mean** ±**SD**	
Number of bottles	1.2 ± 0.8	1 [1–2]	1.3 (0.8)	0.8 (0.6)	< 0.01
Number of instillations	2.1 ± 1.1	1 [1–3]	2.2 (1.1)	1.7 (0.8)	< 0.01
Number of previous surgeries	0.7 ± 0.9	1 [0–1]	0.8 (0.9)	0.3 (0.5)	< 0.01

No surgery had been performed on half of the eyes, 32% had one (three eyes submitted to the Express implantation alone, the others submitted to phacoemulsification alone), 13% had two surgeries (mainly a combined phaco-trabeculectomy, twelve eyes submitted to phaco-MIGS), 4% had three surgeries, and two eyes had four surgeries; confirmed glaucomas were treated with more therapies and had been submitted more frequently to surgeries (Table 2).

About clinical data, considering both eyes, the mean±SD (min-max) visual acuity was 7.7 ± 3.3 (0–10) decimals, the mean intraocular pressure (IOP) was 15 ± 5 (6–50) mmHg, the mean visual field index (VFI) value was 81.3 ± 26 (0–100) %, the MD was −6.7 ± 8.1 (−32.2–4.9) dB, and PSD 4.7 ± 3.7 (1.1–14.8) dB. Confirmed glaucoma patients presented worst visual field defect and visual acuity and higher IOP values (Table 1).

**Figure 1:**
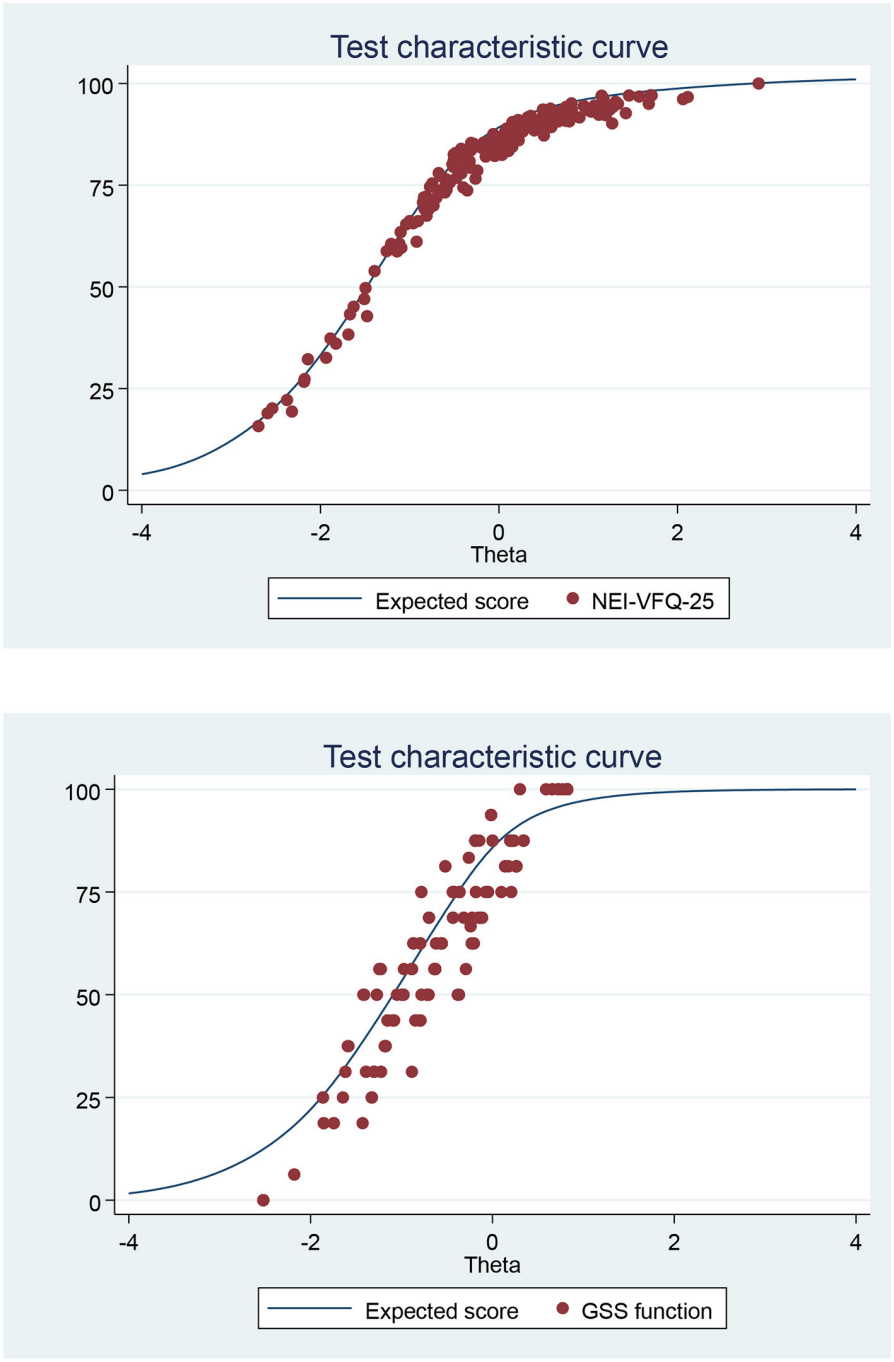
Test characteristic curve (TCC): of NEI-VFQ-25 or GSS summated score vs. predicted score from the graded response model (IRT analysis) of the Italian version of the NEI-VFQ-25 or GSS.

Table 3 presents the scores of both questionnaires: the total mean scores were 80.4 ± 17.8 and 77.2 ± 21 for NEI-VFQ 25 and GSS, respectively. General vision and general health had the worst VRQoL scores. VRQoL was significantly different between suspected and confirmed glaucoma patients apart from color vision and ocular pain scales for NEI-VFQ but were significantly different only for the function scale for the GSS questionnaire (Table 3). Figure 1 shows the strong agreement between the QoL scores calculated according to the questionnaire decoding instructions and those calculated with the IRT-GRM.

**Table 3:** Scores of the two quality of life questionnaires: the 25-item National Eye Institute visual functioning questionnaire (NEI-VFQ 25) and the Glaucoma Symptom Scale (GSS).

**NEI-VFQ 25**	**Overall group**	**Confirmed glaucoma**	**Suspected glaucoma**	***p*-value**
	**Mean** ±**SD**	**Mean** ±**SD**	**Mean** ±**SD**	
General health - GH	57.7 ± 18.3	56 ± 18.5	64 ± 16.4	< 0.01
General vision - GV	60.6 ± 17.4	58 ± 17.3	68 ± 16	< 0.01
Ocular pain - OP	77 ± 21.5	76 ± 22.4	81 ± 17.5	0.190
Near activities - NA	80.7 ± 20.6	80 ± 21	87 ± 15	0.03
Distance activities - DA	86.4 ± 19.3	84.6 ± 21	93 ± 10	0.01
Vision-specific social functioning - VSSF	93.6 ± 16	92.4 ± 14.4	99 ±4	0.02
Vision-specific mental health - VSMH	73.1 ± 23.8	71 ± 25	79 ± 18	0.051
Vision-specific role dependency - VSRD	86.3 ± 22.9	84 ± 25	94 ± 11	0.01
Vision = specific dependency - VSD	88.5 ± 24	86 ± 26	95.5 ± 15	0.03
Driving - D	74 ± 32.3	71.4 ± 34.4	86 ± 16	0.02
Color vision - CV	94 ± 17.3	93 ± 19	98 ± 6.5	0.067
Peripheral vision - PV	86.6 ± 21.2	84.5 ± 23	93.4 ± 12.4	0.02
Total MEAN	80.4 ± 17.8	78.5 ±19	87 ± 10.4	< 0.01
**GSS**				
GSS total	77.2 ± 21	76 ± 22	80.4 ± 17	0.091
GSS symptoms	76.2 ± 23.4	76 ± 24.5	77.4 ± 19	0.644
GSS function	79.1 ± 23.8	76 ±26	85 ± 20	< 0.01

### Univariate analysis

The linear regression univariate analysis has examined the association between QoL questionnaires and different clinical and demographic variables (Table 4).

**Table 4:** Linear regression univariate analysis of the association between the variables analyzed and the mean score of both the NEI-VFQ 25 and the GSS questionnaire (*P*-values and regression coefficients, gende*r* = male).

**Variables**	**Coef**.	***P* > | t |**	**[95% conf. interval]**
**NEI–VFQ25**			
VFI mean	0.33	< 0.001	0.21–0.44
VFI min	0.19	< 0.001	0.11–0.27
VFI max	0.41	< 0.001	0.27–0.55
Gender	3.75	0.146	−1.31–8.81
Age at visit	−0.30	0.016	−0.54 to −0.05
Age at diagnosis	0.13	0.222	−0.07 to −0.34
Years of diseases	−0.32	0.02	−0.58 to −0.05
Visual acuity min	2.03	< 0.001	1.45 to −2.62
Visual acuity max	3.96	< 0.001	2.96 to −4.97
Comorbidities	−0.57	0.546	−2.45–1.3
POAG	−2.2	0.408	−7.44–3.04
IOP min	1.12	0.002	0.41–1.85
IOP max	0.38	0.21	−0.22–0.99
Previous surgeries	−3.94	< 0.001	−5.57 to −2.1
Topical medications	−1.03	0.266	−2.85–0.79
Number of instillations	−0.74	0.179	−1.84–0.35
**GSS**			
VFI	0.41	< 0.001	0.30–0.51
Age at visit	−0.07	0.636	−0.37–0.23
Age at diagnosis	0.25	0.095	−0.04–0.55
Years of disease	−0.19	0.352	−0.62–0.22
Visual acuity	2.37	< 0.001	1.37–3.38
Comorbidities	0.54	0.617	−1.59–2.69
IOP	0.32	0.449	−0.51–1.16
Previous surgeries	−7.27	< 0.001	−10.83 to −3.71
Number of bottles	−3.61	0.087	−7.74–0.52
Number of instillations	−3.66	0.031	−7 to −0.33
Gender	3.37	0.307	−3.13–9.89
Diagnosis	−4.39	0.185	−10.91–2.12

First, the association between the NEI-VFQ 25 mean score and the mean VFI of each patient (VFI mean, i.e., the average between the right and left eye) was analyzed, then the VFI of the worst eye (VFI min) and the VFI of the best eye (VFI max). The VFI is always correlated with the questionnaire mean score and thus the quality of life of the patient (*p* < 0.001). Since the regression coefficient is positive in all three cases, an increase in VFI mean, max, and min is always associated with an increase in the mean score of NEI-VFQ 25 questionnaire, so patients' QoL. A Pearson r of 0.39 is observed with VFI-max.

Regarding other variables, the analysis pointed out that gender is not significantly related to NEI-VFQ mean score while both the age at the time of visit and the time since diagnosis (length of illness) are (*p* = 0.016, inverse relation and *p* = 0.02, direct relation, respectively).

The minimum and maximal visual acuity are significantly correlated with the mean NEI score (*p* < 0.001). On the contrary, comorbidities and the type of diagnosis (POAG vs. the other sub-types of glaucomas) are not correlated with NEI-VFQ (*p* = 0.546 in the former, *p* = 0.408 in the latter). Regarding ocular characteristics, the IOP (min and max) and the number of previous surgeries are related (*p* = 0.002, *p* = 0.017 and *p* < 0.001), while there is no relation with the number of topical medications and the number of instillations of eye drops a day.

Regarding the GSS questionnaire, since the GSS provides two different scores, one for each eye, the VFI is not considered as mean, minimum, and maximum value, but separately. The linear regression analysis (Table 4) pointed out that GSS score does correlate to visual acuity (*p* < 0.001) and VFI (*p* < 0.001), with a positive association, while there is a negative regression coefficient with previous surgeries in the eye considered (*p* < 0.001) and number of instillations (*p* = 0.031). This negative association is also present for the number of bottles and instillations used in the eye, meaning that the GSS function score decreases as the number of bottles of eye drop, instillation, and previous surgeries increases.

### Multivariate analysis

The multivariate analysis found that the VFI max is positively correlated to the QoL: generally, the presence of a higher VFI has a positive impact on the NEI-VFQ score.

About NEI -VFQ 25 questionnaire, gender (*p* = 0.019), visual acuity max (*p* < 0.001), and previous surgeries (*p* = 0.032) have a significant impact: even correcting the association with all these independent variables, there is still a significant association between the VFI max and the QoL examined with NEI-VFQ in the overall group and in the confirmed glaucoma patients (*p* = 0.006 and *p* = 0.028, respectively) (Table 5).

**Table 5:** Multivariate analysis of the association between the VFI, NEI-VFQ 25 (mean and subscales), and confounding variables (*P*-values and regression coefficients) (for the 12 subscales only the significant data are reported; gender refers to male).

**Variables**	**Coef**.	***P* > | t |**	**[95% conf. interval]**
**Overall group**			
VFI max	0.22	0.006	0.06–0.37
Age at visit	0.04	0.702	−0.17–0.25
Gender	5.12	0.019	0.86– 9.39
Visual acuity max	3.15	< 0.001	2.02–4.29
IOP min	0.08	0.811	−0.57–0.73
Previous surgeries	−1.79	0.032	−3.43 to −0.15
Number of instillations	−0.13	0.77	−1.07–0.79
**Confirmed glaucoma**			
VFI max	0.20	0.028	0.02–0.37
Age at visit	−0.01	0.947	−0.3–0.28
Gender	4.23	0.124	−1.18–9.63
Visual acuity max	3.21	< 0.001	1.93–4.48
IOP min	0.07	0.879	−0.81–0.94
Previous surgeries	−1.72	0.055	−3.69–0.24
Number of instillations	−0.09	0.884	−1.28–1.11
**Suspected glaucoma**			
VFI max	2.13	0.122	−0.6–4.87
Age at visit	0.15	0.356	−0.17–0.47
Gender	8.71	0.027	1.04–16.39
Visual acuity max	2.36	0.214	−1.44–6.16
IOP min	0.25	0.632	−0.8–1.3
Previous surgeries	0.27	0.893	−3.73–4.26
Number of instillations	−0.47	0.588	−2.24–1.29
**NEI–VFQ 25 Total mean Subscales**			
**General health (GH)**			
Age at visit	−0.31	0.018	−0.57 to −0.05
Gender	9.51	< 0.001	4.36–14.65
Visual acuity max	1.76	0.012	0.39–3.13
**General vision (GV)**			
Gender	5.75	0.012	1.27–10.22
Visual acuity max	2.94	< 0.001	1.74–4.13
**Ocular pain (OP)**			
Visual acuity max	2.81	0.011	0.47–3.68
Previous surgeries	−2.68	0.023	−4.99 to −0.37
**Near activities (NA)**			
Visual acuity max	3.61	< 0.001	2.2–5.02
Previous surgeries	−2.98	0.004	−5.01 to −0.95
**Distance activities (DA)**			
VFI max	0.24	0.007	0.06–0.41
Gender	5.2	0.032	0.44–9.97
Visual acuity max	3.31	< 0.001	2.04–4.58
**Vision-specific social functioning (VSSF)**			
VFI max	0.17	0.023	0.02–0.33
Visual acuity max	1.55	0.007	0.43–2.68
**Vision-specific mental health (VSMH)**			
VFI max	0.33	0.004	0.11–0.56
Age at visit	0.35	0.027	0.04–0.66
Gender	6.71	0.033	0.54–12.87
Visual acuity max	3.07	< 0.001	1.43–4.72
Previous surgeries	−3.52	0.004	−5.9 to −1.15
**Vision-specific role dependency (VSRD)**			
VFI max	0.33	0.001	0.13–0.53
Visual acuity max	3.82	< 0.001	2.35–5.3
**Vision-specific dependency (VSD)**			
VFI max	0.32	0.003	0.11–0.54
Visual acuity max	3.57	< 0.001	2.01–5.13
**Driving (D)**			
VFI max	0.45	0.006	0.13–0.77
Visual acuity max	5.48	< 0.001	3.14–7.92
**Color vision (CV)**			
Visual acuity max	1.76	0.004	0.58–2.94
**Peripheral vision (PV)**			
VFI max	0.32	0.004	0.11–0.55
**Confirmed glaucoma**			
**GH**			
Age at visit	−0.36	0.03	−0.68 to −0.03
Gender	8.75	0.01	2.66 to −14.83
Visual acuity max	1.65	0.03	0.21–3.09
**GV**			
Gender	5.54	0.04	0.25–10.82
Visual acuity max	2.78	< 0.001	1.53–4.03
**OP**			
Visual acuity max	1.99	0.03	0.23–3.74
**NA**			
Visual acuity max	3.73	< 0.001	2.19–5.27
Previous surgery	−2.89	0.02	−5.26 to −0.52
**DA**			
VFI max	0.21	0.03	0.02–0.41
Visual acuity max	3.61	< 0.001	2.19–5.02
**VSSF**			
Visual acuity max	1.69	0.01	0.41–2.97
**VSMH**			
VFI max	0.34	0.01	0.1–0.58
Visual acuity max	2.93	< 0.001	1.16–4.7
Previous surgery	−3.66	0.01	−6.38 to −0.94
**VSRD**			
VFI max	0.31	0.01	0.08–0.54
Visual acuity max	3.86	< 0.001	2.18–5.55
**VSD**			
VFI max	0.3	0.02	0.06–0.54
Visual acuity max	3.62	< 0.001	1.89–5.55
**D**			
VFI max	0.47	0.01	0.11–0.83
Visual acuity max	5.22	< 0.001	2.63–7.8
**CV**			
Visual acuity max	1.83	0.01	0.45–3.2
**PV**			
VFI max	0.3	0.02	0.04–0.55

Table 5 shows the significant correlation between variables and each sub-scale; there is a significant correlation between VFI and the following sub-scales both in the overall group and in confirmed glaucoma patients: DA, VSMH, VSRD, VSD, D, and PV.

Sensitivity analysis found that the same parameters are significant using predicted latent trait as a dependent variable (gender *p* = 0.010; visual acuity max *p* < 0.001, previous surgery 0.05 and VFI max *p* = 0.008).

GH was significantly worse in older patients (*r* = −0.3, *p* = 0.018); men had significant better GH, GV, DA, and VSMH, while patients submitted to previous surgeries presented statistically significantly worse scores for OP, NA, and VSMH.

Regarding GSS, the multivariate model (Table 6) found an association between the GSS function and the VFI (*p* < 0.001). No association was present among GSS and age at diagnosis (*p* = 0.172), visual acuity (*p* = 0.071), previous surgeries (*p* = 0.232), type of glaucoma (*p* = 0.514), or number of instillations (*p* = 0.5). Sensitivity analysis found that VFI is significant using predicted latent trait as a dependent variable (*p* = 0.001).

**Table 6:** Multivariate analysis of the association between the VFI, GSS (function score), and confounding variables (*P*-values and regression coefficients).

**Variables**	**Coef**.	***P* > | t |**	**[95% conf. interval]**
**GSS function**			
VFI	0.36	< 0.001	0.17–0.55
Age at diagnosis	0.24	0.172	−0.1–0.59
Visual acuity	1.71	0.071	−0.14 to −3.57
Previous surgeries	−3.05	0.232	−8.08–1.98
Diagnosis	2.56	0.514	−5.19–10.31
Number of instillations	−1.22	0.5	−4.81–2.36
**Confirmed glaucoma**			
VFI	0.35	0.001	0.15–0.55
Age at diagnosis	0.31	0.169	−0.13–0.75
Visual acuity	1.75	0.108	−0.39–3.88
Previous surgeries	−3.39	0.252	−9.23–2.46
Diagnosis	7.95	0.13	−2.4–18.3
Number of instillations	−1.22	0.552	−5.31–2.86
**Suspected glaucoma**			
VFI	0.36	0.45	−0.61–1.33
Age at diagnosis	0.08	0.842	−0.73–0.88
Visual acuity	2.11	0.203	−1.23–5.44
Previous surgeries	0.09	0.984	−10.2–10.4
Number of instillations	1.41	0.782	−9.08–11.91

## Discussion

The present study found that the visual field index (VFI), a statistical parameter of the visual field examination, is directly related to the patients' quality of life examined with specific questionnaires, confirming that the residual visual function is correlated with the patient QoL in patients with confirmed glaucoma, since the higher the VFI the higher the total mean score of the questionnaire, which is comprehensive of general health, general vision, and vision-related QoL.

The VFI was originally developed with the goal of addressing the shortcomings of mean deviation (MD). MD is generally used to determine the overall deterioration of visual field; however, it is not sensitive enough to identify a focal visual field loss and can be affected by cataracts and other ocular diseases. Visual field index is a more recent metric of visual loss; it represents the percentage of ganglion cells left, where 0% corresponds to complete blindness and 100% to a normal visual field, better explaining both peripheral and central visual field loss compared to the other indexes. VFI is a percentage that summarizes the overall visual field status compared to age-adjusted visual fields: this parameter emphasizes the importance of the central field. It is less affected by media opacities (cataracts) and is more accurate than MD for monitoring glaucoma progression (6, 9).

In literature, the relationship between quality of life and visual field index has not been addressed often; there are only a few studies reporting some sort of association (7, 8). In 2011, Sawada et al. (7) reported that VFI correlated with QoL by examining the 25-item NEI-VFQ and that the correlation was better than with MD.

In 2014, Lee et al. (8) found a statistically significant correlation between the reduction in mean binocular VFI and a poorer quality of life (examined with the GQL-15 questionnaire) and that VFI was a better indicator of glaucoma-specific QoL than other parameters (RNFL thickness, IOP, or PSD).

Our study found a significant direct relation of the VFI to some sub-scales regarding distance activities, mental health, dependency, driving, and peripheral vision (for NEI-VFQ 25) and to function (for GSS) on confirmed glaucoma patients, underlying a potential predictive value of this parameter in the measure of QoL in patients with glaucoma.

Apart from the association between QoL and VFI, the present study has also considered other confounding variables that may have a positive or negative effect on the quality of life: the study pointed out that there are some demographic and clinical factors that, besides the visual field index, affect the quality of life of glaucoma patients.

The impact of gender on quality of life in glaucoma is not a widely described concept. The results of our study showed that male patients had a significantly higher vision-related quality of life: these results are in agreement with a previous observation made by the Collaborative Initial Glaucoma Treatment Study that found that younger participants and women were more likely to report a decrease in vision-related QoL (10). Apart from the psychological difference between men and women in reacting to the diagnosis of a chronic disease such as glaucoma, the desire to learn about the disease and therefore to understand its potential impact on daily life is also different in the two sexes. In our previous work (11), we observed how knowledge of the disease is related to the type of reaction to the diagnosis in a different way based on gender: women were more interested in learning about prevention, evolution, and causes of glaucoma and, after the diagnosis, developed a reaction of anxiety and fear of blindness.

The age at the time of visit and the total amount of years of illness are significantly related to the quality of life with an indirect relation. A possible explanation for this could be that, since glaucoma is a chronic and progressive disease, older people usually present lower VFI scores due to the disease itself and to the physiological loss of visual function with age (12). Regarding visual acuity, patients with glaucoma may have a very good central visual acuity preserved until terminal stages (tubular defect), therefore the visual acuity of both the worst and the best eye is directly related to higher QoL.

Previous literature has not reached an agreement as to whether it is more important the visual acuity (VA) of the better or of the worst eye: the question remains controversial (10, 13–17).

In 2005, Hyman et al. (16) analyzed the VA of worse and better eyes simultaneously using a multiple linear regression model and has concluded that the VA of the worse eye is more important for VRQoL.

In 2013, Murata et al. (17) suggested that worse-eye VA tended to have a greater impact on VRQoL than better-eye VA; but these results cannot be directly compared to previous reports because, in this study, better and worse-eye VA were defined using MD values, and not VA directly.

Our study confirmed these previous observations, showing that better VA was significantly related to almost all the sub-scales of the NEI-VFQ 25 questionnaire, in particular to general health, general vision, ocular pain, near activities, distance activities, social functioning, mental health, dependency, driving, and color vision. Regarding the GSS questionnaire, there was a slight association only to function scale. Therefore, our data found a positive relation between better visual acuity and better quality of life scores, both general and glaucoma-specific.

Previous literature (18–21) underlined that the deterioration of QoL in early glaucoma is related to the adverse effects, the inconvenience and cost of anti-glaucoma medications, the psychological burden of suffering from a potentially blinding disease, and the alteration of certain aspects of visual function beyond retinal sensitivity, such as color perception, contrast sensitivity, and motion perception. Our data found something different: the type of medical therapies is irrelative and not associated to QoL: a possible explanation could be that side effects due to therapies are less important than the disease itself, but in the present study tolerability and safety of the therapy have not been specifically and directly evaluated.

The present study found a positive association between well-controlled intraocular pressure values (min-IOP) and QoL: a possible explanation and interpretation of this data could lie in the patient's possible thought of keeping the disease under control when they receive a low intraocular pressure value, but this is only a hypothesis and has not been further investigated. Usually, patients know that IOP plays an important role in the control of glaucoma progression, therefore their anxiety decreases when IOP is well controlled (medically or surgically), reaching target pressure, and their QoL increases. Moreover, when IOP is well controlled, the visual field does not worsen also, improving QoL perception (22). In line with previous considerations, our data pointed out that the maximum IOP is negatively associated with NEI-VFQ 25 score.

The number of previous surgeries negatively impact on QoL, probably due to the occurrence of local eye symptoms, such as eye pain, eye redness, and tearing, reported especially in the first 5 years after surgery, as described in the CIGTS study (23). Of note, surgery often represents the last therapeutic option in patients with glaucoma, and patients submitted to surgery usually present more severe visual field defect and lower VFI.

A brief comment on “glaucoma suspects”: our data found no association between VFI and QoL in this subset of patients, but it should be noted that the subset was small and the study was not designed to record any difference between these two types of subjects (confirmed vs. suspected glaucoma). However, this observation deserves further investigation as it has been generally recognized and described that the diagnosis of glaucoma alone has a negative impact on quality of life; our data indicate that the diagnosis of “suspected glaucoma” does not alter the perception of life quality.

## Conclusion

The present study pointed out that visual field index is a parameter that could be useful in clinical practice to score the impact of confirmed glaucoma disease on quality-of-life perception.

To the best of our knowledge, this is one of the few studies reporting a significant correlation and a linear relationship between VFI and the NEI-VFQ 25 in glaucoma patients, and the first evaluating the relationship between VFI and GSS.

Our study demonstrates that the association between visual function (VFI) and QoL does exist, even when considering individual factors and variables, underlying that QoL is not an objective measurement, but a complex subjective parameter.

The study found that VFI has a direct association with vision-related QoL examined with both a generic and glaucoma-specific QoL questionnaire, thus, the visual field index could become an important metric instrument in the follow up of patients with glaucoma, representing a very reliable parameter to assess vision-related QoL.

More data should be analyzed to verify if VFI could become a tool to screen QoL perception in glaucoma patients.

## Data availability statement

The raw data supporting the conclusions of this article will be made available by the authors, without undue reservation.

## Author contributions

GCMR conceptualized and wrote the main manuscript text. ADS and GG performed the statistical analysis and prepared the figures. GCMR, LS, and CB collected the data, entered the data into the database, and prepared tables and figures. All authors reviewed and approved the manuscript.
